# Implementation of Robotic-Assisted Surgery for the Treatment of Patients with Endometrial Carcinoma

**DOI:** 10.3390/cancers17193097

**Published:** 2025-09-23

**Authors:** Walid Shaalan, Kathrin Haßdenteufel, Fabiola Hoppe, Peter Sinn, Riku Togawa, Lara Meike Tretschock, Dina Batarseh, Helmi Ylitalo, Nourhan Hassan, Benedikt Schäfgen, Andre Hennigs, Katharina Smetanay, Andreas Schneeweiss, Lisa Katharina Nees, Fabian Riedel, Oliver Zivanovic

**Affiliations:** 1 Department of Gynecology and Obstetrics, Heidelberg University Hospital, Im Neuenheimer Feld 440, 69120 Heidelberg, Germany; kathrin.hassdenteufel@med.uni-heidelberg.de (K.H.); larameike.tretschock@med.uni-heidelberg.de (L.M.T.); dina.batarseh@med.uni-heidelberg.de (D.B.); benedikt.schaefgen@ukmuenster.de (B.S.); andre.hennigs@med.uni-heidelberg.de (A.H.); lisakatharina.nees@med.uni-heidelberg.de (L.K.N.); fabian.riedel@med.uni-heidelberg.de (F.R.); 2 National Center for Tumor Diseases (NCT), University Hospital Heidelberg, Im Neuenheimer Feld 440, 69120 Heidelberg, Germany; fabiola.hoppe@med.uni-heidelberg.de (F.H.); riku.togawa@med.uni-heidelberg.de (R.T.); helmi.ylitalo@stud.uni-heidelberg.de (H.Y.); katharina.smetanay@med.uni-heidelberg.de (K.S.); andreas.schneeweiss@med.uni-heidelberg.de (A.S.); 3 Institute of Pathology, Heidelberg University, Im Neuenheimer Feld 224, 69120 Heidelberg, Germany; peter.sinn@med.uni-heidelberg.de; 4 Center for Molecular Medicine Cologne, University of Cologne, Robert-Koch-Straße 21, 50931 Cologne, Germany; nourhan.hassan@uk-koeln.de; 5 Biotechnology Department, Faculty of Science, Cairo University, Giza 12613, Egypt; 6 Department of Gynecology and Obstetrics, Münster University Hospital, Albert-Schweitzer Street 33, 48149 Münster, Germany

**Keywords:** endometrial cancer, robotic surgery, conventional laparoscopy, sentinel lymph node mapping, postoperative outcomes, surgical oncology

## Abstract

**Objective:** This retrospective cohort study compares surgical outcomes among patients with endometrial carcinoma (EC) after the implementation of a robotic-assisted (RA) surgical program at a tertiary care center. **Methods:** A total of 122 EC patients who underwent surgery between March 2022 and February 2025 were included. Patients were divided into two cohorts based on the implementation of RA surgery: Group 1 (March 2022–August 2023) and Group 2 (September 2023–February 2025). Data collected included demographics, surgical approach, operative time, hospital stay, completion of staging procedures, and 30-day postoperative complications. **Results:** RA laparoscopy was used predominantly in Group 2, replacing conventional laparoscopy (CL). Laparotomy was significantly less frequent in group 2 (11.9% vs. 36.4%; *p* < 0.001). Among patients with FIGO stage I, all patients underwent minimally invasive surgery (MIS) in Cohort 2 (100% vs. 71.9%; *p* < 0.001). Median hospital stay was significantly shorter in Group 2 (3 days vs. 4 days; *p* < 0.001). A 30-day mortality occurred in one patient (*n* = 1) within the total study cohort (0.82%) and was attributed to pulmonary embolism on postoperative day 14 after RA laparoscopy. Rates of Grade ≥3 postoperative complications were similar (7.3% vs. 7.5%), as were wound complications (5.5% vs. 3%). The use of sentinel lymph node (SLN) mapping increased significantly in Group 2 (91% vs. 54.5%; *p* < 0.001). Completion staging procedures were significantly reduced in group 2 (9.1% vs. 0%; *p* = 0.017). **Conclusions:** The integration of RA laparoscopy significantly reduced laparotomy rates and hospital stays while increasing SLN mapping. These results support the continued adoption of RA laparoscopy to enhance MIS and improve patient outcomes.

## Simple Summary

This retrospective cohort study evaluated the impact of robotic-assisted surgery implementation on endometrial carcinoma outcomes at a tertiary care center. Among 122 patients treated across two periods (March 2022–February 2025), the introduction of robotic surgery in September 2023 significantly transformed surgical practice. Key findings demonstrated a 67% reduction in laparotomy rates (36.4% to 11.9%, *p* < 0.001), complete elimination of laparotomy in FIGO Stage I disease, and a 25% reduction in hospital stay (4 to 3 days, *p* < 0.001). Sentinel lymph node mapping success improved dramatically from 54.5% to 91.0% (*p* < 0.001), while completion staging procedures were eliminated entirely (9.1% to 0%, *p* = 0.017). Complication rates remained comparable between groups, confirming safety during the learning curve. These findings support robotic surgery adoption for enhancing minimally invasive capabilities and optimizing perioperative outcomes in endometrial carcinoma management.

## 1. Introduction

Endometrial cancer is the most common gynecologic malignancy in developed countries and the sixth most frequently diagnosed cancer among women worldwide. In 2018 alone, there were an estimated 382,069 new cases and 89,929 deaths attributed to this disease [1,2]. While endometrial carcinoma predominantly affects postmenopausal women, its global incidence has been steadily rising, particularly among older age groups and individuals with high body mass index (BMI). This trend has heightened the need to optimize treatment strategies and improve outcomes for patients with endometrial carcinoma [3,4].

Fortunately, the majority of endometrial carcinoma cases are diagnosed at an early stage, when the disease is confined to the uterus. This early detection contributes to high survival rates [5,6]. Surgical intervention remains the cornerstone of management for early-stage endometrial carcinoma, typically involving total hysterectomy, bilateral salpingo-oophorectomy, and pelvic and paraaortic lymphadenectomy [7].

The advent of minimally invasive surgery (MIS) has revolutionized the management of endometrial carcinoma (EC). Landmark studies, such as the Gynecologic Oncology Group (GOG)-LAP2 trial and the Laparoscopic Approach to Cancer of the Endometrium (LACE) trial, have demonstrated that minimally invasive surgery (MIS) offers oncologic outcomes equivalent to those of laparotomy while significantly reducing perioperative morbidity, hospital stays, and recovery times [8,9,10]. These findings have solidified minimally invasive surgery (MIS) as the preferred surgical approach for endometrial carcinoma on a global scale [11]. Nevertheless, the technically demanding nature of conventional laparoscopy (CL) can pose challenges, particularly in obese patients and complex or high-risk situations where advanced surgical skills are required [12]. This is reflected in the higher conversion rates to laparotomy observed in the LAP2 trial.

Robot-assisted (RA) laparoscopy has emerged as a promising alternative. Robotic-assisted (RA) surgery offers enhanced precision, three-dimensional visualization, and improved instrument dexterity, making it particularly advantageous for obese patients and complex procedures [13,14,15]. Additionally, robotic surgery is associated with shorter hospital stays, reduced blood loss, and faster postoperative recovery [16,17]. Studies also suggest that robotic-assisted (RA) surgery may have a shallower learning curve and greater applicability in obese or elderly patients, who often present unique surgical challenges [18,19]. Despite these benefits, the high acquisition and maintenance costs of robotic systems, along with the lack of tactile feedback, remain significant barriers to widespread adoption. Furthermore, the increased setup time and limited availability of robotic systems in certain areas of the world further complicate their accessibility [20,21].

The implementation of robotic-assisted surgery in gynecologic oncology represents more than a simple technological upgrade; it constitutes a paradigm shift that can fundamentally alter surgical outcomes and healthcare delivery. Recent systematic reviews and meta-analyses have demonstrated that robotic surgery not only maintains the oncologic safety of conventional minimally invasive approaches but may offer superior perioperative outcomes, particularly in complex cases and challenging patient populations [22,23].

From a healthcare systems perspective, the adoption of robotic surgery requires careful evaluation of multiple factors, including clinical outcomes, cost-effectiveness, learning curve considerations, and impact on surgical training programs. The German healthcare system, with its emphasis on evidence-based medicine and cost-effective care delivery, provides an ideal setting to evaluate the real-world impact of robotic surgery implementation [24].

The successful integration of robotic surgery into clinical practice requires systematic evaluation of quality metrics beyond traditional surgical outcomes. Key performance indicators include conversion rates to laparotomy, completion of staging procedures, adherence to evidence-based surgical guidelines, and patient-reported outcomes. Additionally, the learning curve associated with robotic surgery implementation necessitates careful monitoring of outcomes during the transition period to ensure patient safety is maintained while surgical teams develop proficiency [25,26].

While existing literature supports the benefits of robotic surgery in endometrial carcinoma, several important questions remain unanswered. The optimal patient selection criteria for robotic versus conventional laparoscopic approaches are not well-defined. Additionally, the economic impact of robotic surgery implementation, particularly in European healthcare systems, requires further investigation. Most importantly, real-world implementation studies that capture the complete transition experience, including learning curve effects and system-wide changes, are limited in the literature [27,28].

In September 2023, we introduced a robotic program at our department, including standard training procedures and proctoring of faculty. This implementation followed a comprehensive, evidence-based framework designed to ensure optimal outcomes while minimizing risks associated with new technology adoption. Our approach included systematic surgeon training and certification, multidisciplinary team education, standardized protocols for patient selection and surgical technique, and rigorous outcome monitoring throughout the implementation period.

The timing of our implementation coincided with updated international guidelines from ESGO (European Society of Gynaecological Oncology) that increasingly emphasized the role of sentinel lymph node mapping and minimally invasive surgical approaches in endometrial carcinoma management. This provided an opportunity to evaluate not only the direct impact of robotic technology but also the concurrent adoption of evidence-based surgical practices [7].

This study aims to compare outcomes between two distinct time periods based on the implementation of robotic-assisted (RA) surgery in the management of endometrial carcinoma (EC), with a focus on perioperative outcomes among patients undergoing surgery for endometrial carcinoma (EC). Specifically, we hypothesized that the systematic implementation of robotic-assisted surgery would result in (1) reduced rates of conversion to laparotomy, particularly in early-stage disease; (2) improved adherence to evidence-based staging procedures, including sentinel lymph node mapping; (3) enhanced perioperative outcomes, including reduced hospital stays and maintained safety profiles; and (4) improved surgical quality metrics, including reduced need for completion staging procedures.

This comprehensive evaluation aims to provide evidence-based insights into the impact of robotic surgery implementation in a tertiary gynecologic oncology center, informing future adoption strategies and contributing to the growing body of literature on surgical innovation in cancer care. Our findings will be particularly relevant for healthcare systems considering robotic surgery implementation and for clinicians seeking to optimize surgical outcomes in endometrial carcinoma management.

This study aims to compare surgical outcomes before and after the implementation of robotic-assisted (RA) surgery in patients with endometrial carcinoma (EC). By providing a comprehensive analysis of this technological transition, we aim to contribute valuable real-world evidence to guide clinical decision-making, healthcare policy development, and future research priorities in gynecologic oncology.

## 2. Patients and Methods

### 2.1. Study Population and Data Collection

This retrospective observational cohort study included 122 consecutive patients diagnosed with endometrial carcinoma who underwent surgical treatment at Heidelberg University Hospital (Heidelberg, Germany) between March 2022 and February 2025. The surgical procedures performed consisted of total hysterectomy and bilateral salpingo-oophorectomy, with or without lymph node assessment. Lymph node assessment was performed either using the sentinel lymph node algorithm [22] or using a systematic lymph node dissection. The sentinel lymph node (SLN) mapping was conducted uniformly across both study groups, utilizing Indocyanine Green (ICG) (PULSION Medical Systems SE, Feldkirchen, Germany). The Indocyanine Green (ICG) solution, prepared at a concentration of 1.25 mg/mL, was administered by injecting 1 mL deeply into the cervical stroma and 1 mL superficially at the 3- and 9-o’clock positions of the cervix [23].

Patients were identified through a systematic review of institutional surgical and pathological databases. The study was designed as a comprehensive quality improvement assessment following the implementation of a major technological advancement in our department, allowing us to capture the complete patient experience during the transition period, including learning curve effects and system-wide changes. Inclusion criteria comprised histologically confirmed endometrial carcinoma, comprehensive surgical and pathological documentation, and a follow-up period sufficient to assess short-term surgical outcomes. Patients with incomplete medical records or contraindications to surgery were excluded from the study. Patients who had their hysterectomy performed at an outside hospital were excluded. Patients who underwent minimally invasive surgery (MIS) and in whom intraoperative conversion from minimally invasive surgery (MIS) to laparotomy was necessary (*n* = 1) were classified as the laparotomy group. A mini-laparotomy for the delivery of a specimen was not grouped as laparotomy. Specifically, during the study period, 131 patients with a histological diagnosis of endometrial carcinoma were identified in our institutional database. Of these, 122 patients were included in the final analysis. Specifically, 7 patients were excluded because the primary hysterectomy had been performed at an outside institution, and 2 patients were excluded due to contraindications to surgery related to severe multimorbidity.

The following variables were collected: patient demographics (age, body mass index (BMI)), preoperative Eastern Cooperative Oncology Group (ECOG) performance status, and surgical approach (robotic-assisted (RA), conventional laparoscopy (CL), or laparotomy). Surgical details, including operative time, sentinel lymph node (SLN) mapping, lymph node yield, and the necessity for completion staging procedures, were recorded. Postoperative outcomes, including length of hospital stay and 30-day mortality and morbidity, were also documented [24]. Adverse events were graded according to the Clavien–Dindo surgical event system. Only Grade 3 or higher were considered, with the exception of wound complications. Tumor characteristics, such as the International Federation of Gynecology and Obstetrics (FIGO) stage 2009, histopathological subtype, molecular classification, and tumor grading, were assessed based on histopathological and molecular diagnostic data.

### 2.2. Data and Statistical Analysis

Electronic medical records (EMRs) served as the primary source for collecting patient information, including demographic details (e.g., age, body mass index (BMI)), clinicopathologic features (e.g., tumor stage, histology), and 30-day postoperative outcomes. To ensure accuracy, the study team cross-verified all data points against original clinical reports, pathology results, and follow-up visit documentation.

Data were analyzed using IBM SPSS software version 20.0 (Armonk, NY, USA: IBM Corp.) and GraphPad Prism version 10.0 for advanced statistical comparisons. Descriptive statistics were calculated to summarize demographic, surgical, and postoperative variables. Continuous variables, such as age, body mass index (BMI), and hospital stay duration, were reported as means with standard deviations or medians with interquartile ranges, depending on the distribution of the data. Categorical variables, including surgical approach, International Federation of Gynecology and Obstetrics (FIGO) stage, histopathological subtype, and molecular classification, were presented as frequencies and percentages.

To evaluate differences among patient subgroups, appropriate statistical tests were applied. Continuous variables with normal distributions were analyzed using Student’s *t*-tests, while non-normally distributed data were assessed with Mann–Whitney U tests. Categorical variables were compared using chi-square tests or Fisher’s exact tests for small sample sizes. The Kruskal–Wallis test was used for comparisons involving more than two groups, with post hoc (Dunn’s multiple comparison test) for pairwise comparisons. To address potential confounding variables and establish independent effects of robotic surgery implementation, we performed comprehensive multivariate regression analyses. Logistic regression was used for binary outcomes and linear regression for continuous outcomes. All *p*-values were two-sided, with statistical significance set at alpha <0.05.

Ethical approval for the study was granted by the Institutional Review Board at Heidelberg University Hospital (IRB Approval Number: S-031/2025), and data collection followed the principles outlined in the Declaration of Helsinki and STROBE guidelines for observational studies.

## 3. Results

A total of 122 patients were included in the study: 55 patients in Group 1 (treated between March 2022 and August 2023) and 67 patients in Group 2 (treated between September 2023 and February 2025). The patient characteristics are listed in Table 1 and Table 2. No significant differences were observed in the median age of patients, body mass index (BMI), or Eastern Cooperative Oncology Group (ECOG) performance status.

**Figure 1:**
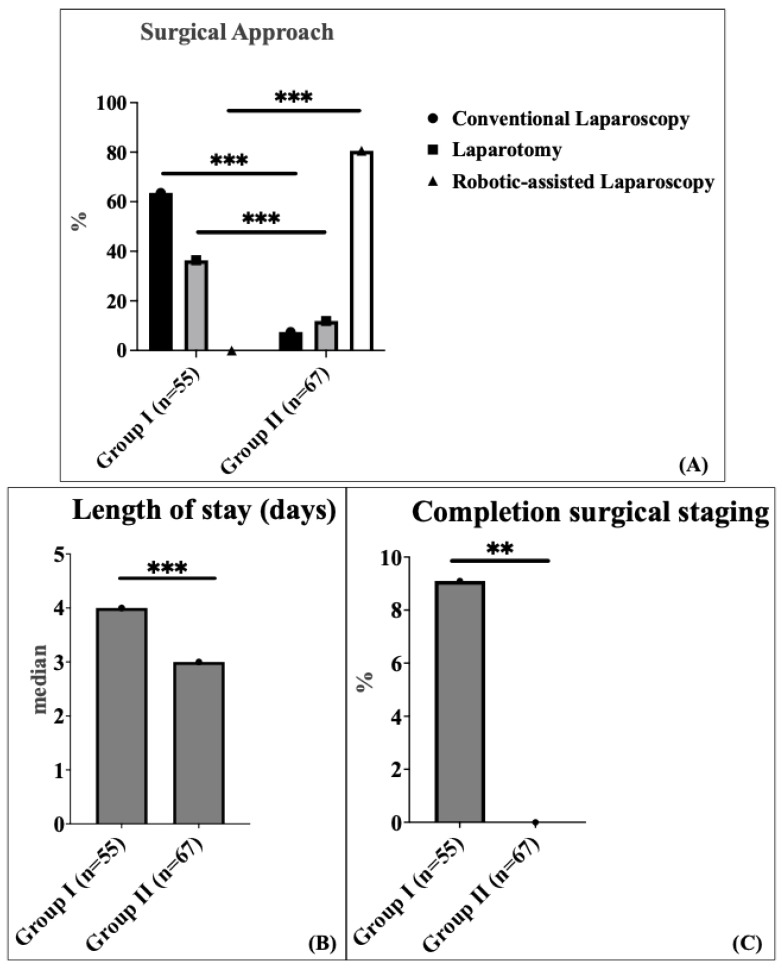
Schematic representation of the distribution of surgical approaches (Black: conventional laparoscopy, grey: laparotomy, white: robotic-assisted laparoscopy) (**A**), length of stay (days) (**B**), and completion of surgical staging (**C**) in endometrial cancer patients. Asterisks (*** and **) denote statistical annotations that indicate statistical significance (*p* < 0.001 and *p* < 0.01), respectively. Black: conventional laparoscopy, grey: laparotomy, white: robotic-assisted laparoscopy.

The surgical approach varied significantly between the two groups. In Group 1, conventional laparoscopy (CL) was the most common method, performed in 63.6% of patients, followed by laparotomy in 36.4% of patients. In contrast, Group 2 predominantly utilized robotic-assisted (RA) laparoscopy, which accounted for 80.6% of patients, while laparotomy and conventional laparoscopy (CL) were performed in 11.9% and 7.5% of patients, respectively (*p* < 0.001) (Figure 1A).

There was no significant difference in median operating time between the two groups: 144 min (interquartile range (IQR) 115.5–213.5) in Group 1 compared to 167 min (interquartile range (IQR) 141.0–206.5) in Group 2. The comparison was performed using the Mann–Whitney U test (non-parametric test). The 23-min increase in median operative time (95% CI: −36.0 to 5.0 min) likely reflects the well-documented learning curve associated with robotic surgery implementation. The median length of hospital stay was significantly shorter in Group 2 (3 days; interquartile range (IQR) 2.0–3.0) compared to Group 1 (4 days; interquartile range (IQR) 3.0–7.5) (*p* < 0.001) (Figure 1B). This represents a 25% reduction in hospital stay with a robust confidence interval (95% CI: 1.0–2.0 days), demonstrating consistent benefit across the patient population. Completion surgical staging was defined as a secondary procedure to complete lymph node assessment after an initial hysterectomy with bilateral salpingo-oophorectomy. This applied to five patients (9.1%) in Group 1 and none (0%) in Group 2, following the definition established by Concin et al., 2021 [1]. Completion surgical staging after incompletely staged endometrial carcinoma was used significantly more frequently in Group 1 (9.1%) compared to Group 2 (0%) (*p* = 0.017) (Figure 1C).

To address potential confounding variables, we performed comprehensive multivariate regression analyses adjusting for age, BMI, ECOG performance status, FIGO stage, and histological subtype. The findings of the univariate analyses remained statistically significant after performing the multivariate analyses, confirming the independent effect of robotic surgery implementation (Table 3). This direct comparison demonstrates that robotic surgery provides significant advantages over conventional laparoscopy in hospital stay reduction (25% decrease) and sentinel lymph node mapping success (98.1% vs. 80.0%), while maintaining comparable safety profiles despite longer operative times during the learning curve period.

Major complications (Clavien–Dindo 3 or higher) in the early postoperative period (within 30 days) were not significantly different between cohort 1 (7.3%) and cohort 2 (7.5%). In detail, we recorded four early surgery-related complications in Group 1 (three patients with vaginal cuff hematoma and one urinary bladder injury, all of which required surgical revision). In Group 2, a total of five complications were recorded (two patients experienced bowel injuries, both with a history of complex abdominal surgeries, including multiple bowel resections for prior sigmoid carcinoma). One patient developed a Hartmann’s cuff insufficiency with an associated vaginal cuff hematoma; this patient had undergone prior radiochemotherapy for advanced-stage disease before being referred to our center for surgical management, all of which required surgical revision; and two patients developed infected lymphoceles, which required computer tomography (CT)-guided drainage.

Wound infections were comparable between the groups (5% vs. 3.5%). A single 30-day mortality (*n* = 1) occurred within the total study cohort and was reported in Cohort 2 on postoperative day 14 following robotic-assisted (RA) surgery, attributed to pulmonary embolism.

**Figure 2:**
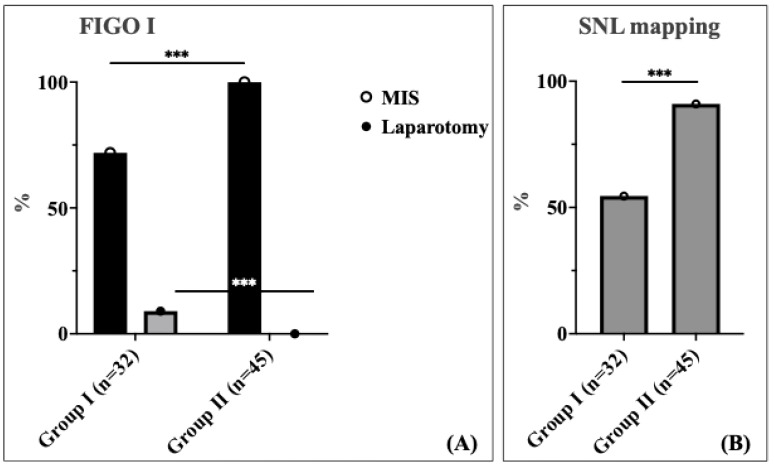
Schematic representation of FIGO stage I endometrial cancer (black: MIS, grey: laparotomy) (**A**), and sentinel lymph node (SLN) mapping (**B**) in endometrial carcinoma. The percentage value (%) indicates the detection rate or nodal involvement. Asterisks (***) denote statistical annotations that indicate statistical significance (*p* < 0.001).

A significant difference was observed in the surgical approach between the two cohorts for patients with FIGO stage I (Figure 2A). In this stage, 28.1% of patients in Cohort 1 underwent laparotomy, whereas no patients in Cohort 2 required laparotomy; all patients in this group were managed using the minimally invasive surgery (MIS) approach (*p* < 0.001). This represents a complete elimination of unnecessary laparotomies in early-stage disease, demonstrating the enhanced precision and capability of robotic-assisted surgery. For FIGO stage II, the distribution of laparotomy rate was comparable between the two cohorts (30.8% vs. 16.7%). Among patients with locally advanced stage III and IV according to the International Federation of Gynecology and Obstetrics (FIGO), the laparotomy rate was 70.0% in Cohort 1 compared to 43.8% in Cohort 2 without statistical significance (Table 4).

The study revealed a comparable distribution of histopathological subtypes between the two groups. Endometrioid adenocarcinoma was the predominant histopathological subtype in both cohorts (Group 1: 94.5%; Group 2: 80.6%), followed by serous carcinoma (Group 1: 3.6%; Group 2: 13.4%). We observed a higher proportion of serous carcinoma in Group II (13.4% vs. 3.6%), although this result was not statistically significant (*p* = 0.109). As allocation was based on treatment period rather than histology, this finding reflects temporal variation within the cohort. To address potential confounding, we performed subgroup analysis excluding serous carcinoma cases, and our primary findings (hospital stay reduction, SLN mapping improvement, and laparotomy rate reduction) remained statistically significant. The grading, International Federation of Gynecology and Obstetrics (FIGO) stage distribution, and molecular classification are listed in Table 5.

Tumor grade, alongside histological subtype and molecular profile, was considered during multidisciplinary tumor board discussions guiding surgical planning. In Group 2, SLN mapping was the preferred strategy for early-stage disease, regardless of grade, in line with international guidelines (ESGO 2021) [1]. Systematic lymphadenectomy was performed for select cases with suspected lymph nodes or failed SLN mapping.

Sentinel lymph node (SLN) mapping was performed significantly more frequently in patients in Group 2 compared to those in Group 1 (91.0% vs. 54.5%, *p* < 0.001) (Figure 2B). The dramatic improvement in SLN mapping rates demonstrates enhanced surgical precision and adherence to evidence-based staging protocols following robotic surgery implementation. We found no statistically significant difference in the total number of lymph nodes (LNs) harvested between the two cohorts. The median number of lymph nodes (LNs) harvested was five in both groups, with an IQR of 2.0–24.0 for Group 1 and 3.0–8.5 for Group 2.

The maintained benefits of robotic surgery across all BMI categories, with pronounced improvements in higher BMI patients, support its value in this challenging patient population and in avoiding laparotomy in complex cases.

## 4. Discussion

**Summary of Main Results:** In this retrospective study, we aimed to compare the surgical outcomes of 122 patients with endometrial carcinoma based on the implementation of robotic-assisted (RA) surgery across two consecutive periods. Baseline demographic and clinical characteristics, including age, International Federation of Gynecology and Obstetrics (FIGO) stage, histology type, body mass index (BMI), and Eastern Cooperative Oncology Group (ECOG) performance status, were comparable between the groups, ensuring a homogeneous study population for the analysis.

Surgical outcomes, such as operative time and lymph node (LN) yield, were consistent across both groups, reflecting surgical proficiency and adherence to standardized protocols. The low and comparable complication rates observed in this study further underscore the safety and reliability of contemporary surgical approaches in a tertiary care setting.

**Results in the Context of Published Literature:** The advantages of robotic-assisted (RA) surgery in terms of shorter hospital stays identified by our study are in line with the literature [25]. The large retrospective study by Casarin et al. [14], analyzing data of 35,224 patients who underwent total hysterectomy for endometrial cancer in US hospitals, supported the finding that the robotic-assisted (RA) surgery results in a shorter hospitalization when compared to the laparoscopic and laparotomic techniques, thereby corroborating our findings.

The observed reduction in hospital stay from a median of 4 days to 3 days represents a 25% decrease that carries substantial clinical and economic implications. While we did not conduct a formal cost-effectiveness analysis within the German healthcare system, existing literature demonstrates significant economic benefits [24].

Furthermore, our findings indicate the potential advancements in perioperative care and enhanced recovery (ERAS) protocols in the latter study group. Recent studies have demonstrated that this approach is associated with higher patient satisfaction, lower overall healthcare costs, and a reduced risk of hospital-acquired complications. Contributing factors likely include optimized perioperative pain management, early postoperative mobilization, and adequate discharge planning [26].

Additionally, completion staging procedures after initial incomplete staging surgery were significantly lower in Group 2, primarily attributed to the increased adoption of sentinel lymph node (SLN) mapping during the latter study period. This trend aligns with emerging evidence highlighting sentinel lymph node (SLN) mapping as an effective strategy for minimizing unnecessary systematic lymphadenectomies, thereby minimizing postoperative morbidity while maintaining oncologic safety. These findings are consistent with the recent study of Bogani et al. [27], underscoring the growing recognition of sentinel lymph node (SLN) mapping as a valuable approach for optimizing surgical management in patients with endometrial carcinoma.

In our study, all patients with FIGO stage I disease in Cohort 2 successfully underwent minimally invasive surgery (MIS), and no patient required a laparotomy. In contrast, a laparotomy was deemed necessary in 28% of patients in Cohort 1. This complete elimination of unnecessary laparotomies in early-stage disease represents a paradigm shift in surgical approach, with major implications for patient outcomes. The 100% minimally invasive surgery achievement in FIGO Stage I disease demonstrates the enhanced precision and capability of robotic-assisted surgery in overcoming technical challenges that previously necessitated conversion to laparotomy. This likely reflects the additional complementary benefit of robotic-assisted surgery and the limitations of conventional laparoscopy in complex situations such as morbid obesity or adhesions due to prior surgeries. This approach aligns with findings from LAP2 and Janda et al., which demonstrated comparable survival outcomes and shorter hospital stays with minimally invasive surgery (MIS) in early-stage endometrial cancer [8,9,10]. These findings reinforce the successful integration of evidence-based practice, emphasizing the feasibility and safety of minimally invasive surgery (MIS) as the preferred approach in early-stage disease.

Although robotic-assisted surgery offers several advantages, the 23-min increase in median operative time (167 vs. 144 min) reflects the well-documented learning curve associated with robotic surgery implementation. This finding contrasts with some studies reporting longer operative times with robotic surgery, but our results suggest that enhanced surgical training using the robotic double-console, streamlined workflows, and standardized training protocols may have mitigated this concern in our setting. Importantly, the operative time difference did not translate into increased complications or adverse outcomes, supporting the safety of robotic surgery implementation when appropriate training protocols are followed.

The learning curve effect is particularly relevant during the initial implementation phase. Literature suggests that robotic proficiency typically requires 20–50 cases, depending on surgeon experience and case complexity. Our implementation occurred in September 2023, meaning many Group 2 cases represent early learning curve experiences. We observed a temporal trend toward shorter operative times in the latter half of Group 2, suggesting progressive improvement with experience [2].

In contrast to our findings, Fu et al. [28] have reported that robotic-assisted (RA) surgery may be associated with a lower lymph node (LN) yield, potentially due to reduced tactile feedback and procedural challenges. However, our results emphasize the reliability of robotic-assisted (RA) surgery in achieving effective oncologic staging.

Although RA surgery offers several advantages, a large study by Perrone et al. [24] has reported longer operative times compared to conventional laparoscopy (CL), potentially attributable to the initial learning curve associated with the technique. In our study, operative time did not differ significantly between the two cohorts, suggesting that enhanced and formalized surgical training using the robotic double-console, streamlined workflows, and training protocols may have mitigated this concern in our setting.

Our comparison between RA surgery and conventional laparoscopy (excluding laparotomy cases) provides crucial insights into the specific advantages of robotic technology beyond simply avoiding laparotomy in complex patients. The 98.1% sentinel lymph node mapping success rate with robotic surgery vs. 80.0% with conventional laparoscopy demonstrates superior technical precision, likely due to enhanced visualization and instrument dexterity. Even when comparing only minimally invasive approaches, robotic surgery achieved significantly shorter hospital stays, suggesting benefits beyond simply avoiding laparotomy. This analysis demonstrates that robotic surgery provides incremental benefits over conventional laparoscopy, particularly in surgical precision and recovery optimization. While robotic surgery involves higher equipment costs, the demonstrated benefits in hospital stay reduction and improved surgical precision may offset these costs through reduced readmissions, fewer completion procedures, and enhanced surgical efficiency over time.

We observed a higher proportion of serous carcinoma in Group II (13.4% vs. 3.6%), representing a 3.7-fold increase that, while not statistically significant (*p* = 0.109), warrants discussion given the more aggressive nature of serous carcinoma. This temporal variation may reflect natural fluctuations in referral patterns to our tertiary center or increased recognition of our robotic capabilities, leading to more complex case referrals. Importantly, our subgroup analysis excluding serous carcinoma cases confirmed that all primary findings (hospital stay reduction, SLN mapping improvement, and laparotomy rate reduction) remained statistically significant, suggesting that the observed benefits extend across histological subtypes and may be particularly valuable for complex cases.

In the study by Barbash et al. [29], higher costs associated with robotic-assisted (RA) were reported without corresponding improvements in outcomes. In contrast, our findings demonstrated improved perioperative outcomes in the robotic-assisted (RA) cohort, notably in terms of shorter hospital stays, which may be associated with lower healthcare costs. In our study, more patients were amenable to minimally invasive surgery (MIS), effectively reducing the rate of laparotomy. Especially in the setting of obesity, robotic-assisted (RA) surgery is expanding the minimally invasive surgery (MIS) rates and avoiding unnecessary laparotomies and their consequences. Our BMI stratification analysis demonstrated maintained benefits across all weight categories, with pronounced improvements in higher BMI patients, supporting robotic surgery’s value in this challenging population where technical difficulties often necessitate conversion to laparotomy with conventional approaches. This is especially important when considering the high risk of wound complications in this patient population [30]. This finding underscores the importance of conducting comprehensive cost–benefit analyses to accurately assess the long-term value of robotic-assisted (RA) surgery.

The integration of robotic-assisted surgery has contributed to several quality improvements beyond the primary outcomes measured. The complete elimination of completion staging procedures represents a major quality improvement with important implications for patient care, healthcare efficiency, and surgical precision. This improvement reflects enhanced intraoperative decision-making and staging accuracy afforded by robotic technology, combined with increased utilization of evidence-based sentinel lymph node mapping protocols.

The improvement in sentinel lymph node mapping rates demonstrates enhanced adherence to evidence-based surgical guidelines and improved surgical precision. This finding is particularly significant given the growing evidence supporting SLN mapping as the preferred staging method for early-stage endometrial carcinoma, offering equivalent oncologic outcomes with reduced morbidity compared to systematic lymphadenectomy.

**Strengths and Weaknesses:** A major strength of this study lies in its comprehensive analysis of a well-defined cohort managed at a tertiary care center, ensuring consistency in surgical and pathological protocols. Moreover, the comparative evaluation across two distinct time periods enables the identification of advancements in clinical practice after the integration of robotic-assisted (RA) laparoscopy. The systematic, department-wide implementation of robotic surgery, rather than selective case allocation, reduces selection bias inherent in surgeon- or patient preference-driven studies. Our high inclusion rate (93.1% of eligible cases) minimizes selection bias and ensures findings are representative of the broader endometrial carcinoma population.

However, our retrospective design introduces limitations. The single-center setting may limit the generalizability of the findings to other healthcare systems or populations. Furthermore, the relatively short follow-up period constrains the assessment of long-term outcomes, such as recurrence and survival. The learning curve effects observed in operative times may not be representative of centers with different training protocols or surgeon experience levels.

**Implications for Practice and Future Research:** Our findings support the broader adoption of robotic-assisted laparoscopy in endometrial cancer management, particularly for its ability to reduce laparotomy rates and facilitate minimally invasive approaches in complex cases. The demonstrated benefits in surgical precision (SLN mapping), recovery optimization (hospital stay), and quality metrics (completion staging elimination) provide evidence-based support for robotic surgery adoption in gynecologic oncology centers. Future studies should focus on several key areas: (1) long-term oncologic outcomes, (2) formal cost-effectiveness analyses, and (3) learning curve optimization strategies and standardized training protocols.

This transition to robotic surgery should be accompanied by standardized training programs to optimize surgeon proficiency and by continued research into patient selection criteria to maximize the benefits of this technology across diverse healthcare settings. The implementation should follow a systematic approach, including comprehensive surgeon training, multidisciplinary team education, and adherence to established quality metrics to ensure reproducible outcomes.

## 5. Conclusions

This retrospective cohort study underscores the role of robotic-assisted (RA) surgery in the management of patients with endometrial carcinoma (EC). Our study demonstrates that robotic-assisted surgery implementation optimizes specific perioperative outcomes, including (1) surgical approach selection with complete elimination of laparotomy in early-stage disease, (2) hospital resource utilization with a 25% reduction in length of stay, and (3) staging procedure precision with a 67% improvement in SLN mapping success and avoidance of completion staging procedures. These improvements were achieved while maintaining comparable safety profiles during the learning curve period, supporting the continued adoption of robotic-assisted surgery to enhance minimally invasive surgical capabilities and improve patient outcomes in endometrial carcinoma management.

## Figures and Tables

**Table 1:** Comparison of demographic, operative, and postoperative characteristics between 122 women with endometrial cancer in Group I and Group II.

	Group I (*n* = 55)	Group II (*n* = 67)	*p*
**1.** **Age (years)**			
Minimum–Maximum	31.0–95.0	27.0–88.0	0.161
Median	66.0	63.0	
IQR	59.0–73.0	56.0–69.0	
**2.** **Body mass index (BMI) (kg/m^2^)**			
Minimum–Maximum	18.36–76.12	17.80–51.42	0.927
Median	29.41	30.30	
IQR	23.45–33.89	24.13–36.24	
**3.** **Eastern Cooperative Oncology Group (ECOG) performance status**			
0 + 1	48 (87.3%)	64 (95.5%)	0.290
2	5 (9.1%)	2 (3.0%)	
3	2 (3.6%)	1 (1.5%)	
**4.** **Surgical approach**			
Robotic-assisted Laparoscopy	0	54 (80.6%)	<0.001 *
Laparotomy	20 (36.4%)	8 (11.9%)	
Conventional Laparoscopy	35 (63.6%)	5 (7.5%)	
**5.** **Operative time (minutes)**			
Minimum–Maximum	68–414	92–550	0.107
Median	144	167	
IQR	115.5–213.5	141.0–206.5	
**6.** **Length of stay (days)**			
Minimum–Maximum	2.0–50.0	2.0–45.0	<0.001 **
Median	4.0	3.0	
IQR	3.0–7.5	2.0–3.0	
**7.** **Completion surgical staging**	5 (9.1%)	0 (0.0%)	0.017 *
**8.** **30-day Morbidity**	4 (7.3%)	5 (7.5%)	1.000
**9.** **Wound complications**	3 (5.5%)	2 (3%)	0.675

*p*: *p*-value for comparing between the studied groups, significant at *p* ≤ 0.05. *: Statistically significant based on the chi-square test. **: Statistically significant based on the Mann–Whitney test. IQR: Interquartile Range.

**Table 2:** Comparison between 122 women with endometrial cancer in Group I and Group II with 95% Confidence Intervals. *: Statistically significant based on the Mann–Whitney test. **^FE^**: Fisher Exact test.

	Group I (*n* = 55)	Group II (*n* = 67)	*p*	Difference (LL–UL 95% C.I)
**Operative time**	144.0 (115.5–213.5)	167.0 (141.0–206.5)	0.107	−17.0 (−36.0–5.0)
**Length of stay (days)**	4.0 (3.0–7.50)	2.0 (2.0–3.0)	<0.001 *	2.0 (1.0–2.0)
**30-day Morbidity**	4 (7.3%)	5 (7.5%)	^FE^*p* = 1.000	0.20 (−11.27–10.72)
**Wound complications**	3 (5.5%)	2 (3%)	^FE^*p* = 0.657	2.50 (−6.07–12.54)
**Sentinel Lymph Node (SLN) Mapping**	30 (54.5%)	61 (91.0%)	<0.001 *	36.50 (19.98–51.57)

**Table 3:** Analysis for the effect of RA and CL on different outcomes.

	CL in Group 1 (*n* = 35)	RA in Group 2 (*n* = 54)	Univariate		Multivariate ^#^	
			*p*	OR/B (LL–UL 95% C.I.)	*p*	OR/B (LL–UL 95% C.I.)
**Length of stay (days)**	4.09 ± 2.75	2.56 ± 0.66	<0.001 *	−1.530 ^&^ (−2.304–−0.756)	<0.001 *	−1.588 ^&^ (−2.386–−0.790)
**30-day Morbidity**	3 (8.6%)	3 (5.6%)	0.582	0.627 ^@^ (0.119–3.301)	0.622	0.651 ^@^ (0.118–3.591)
**Wound complications**	2 (5.7%)	2 (3.7%)	0.657	0.635 ^@^ (0.085–4.726)	0.475	0.460 ^@^ (0.054–3.878)
**Sentinel Lymph Node (SLN) Mapping**	28 (80.0%)	53 (98.1%)	0.018 *	13.250 ^@^ (1.552–113.153)	0.032 *	10.760 ^@^ (1.231–94.025)

Data expressed in (Mean ± SD) and (%). @: OR: Odd’s ratio. &: B: Unstandardized Coefficients (linear regression). #: adjust Odd’s ratio by age, BMI, and ECOG. C.I.: Confidence interval. LL: Lower limit. UL: Upper Limit. *: Statistically significant at *p* ≤ 0.05.

**Table 4:** Comparison of the surgical approach according to FIGO stage between 122 women with endometrial cancer in Group I and Group II.

	Group I	Group II	^FE^ *p*
**FIGO I**	**(*n* = 32)**	**(*n* = 45)**	
Minimally invasive surgery (MIS)	23 (71.9%)	45 (100%)	<0.001 *
Laparotomy	9 (28.1%)	0 (0%)	
**FIGO II**	**(*n* = 13)**	**(*n* = 6)**	
Minimally invasive surgery (MIS)	9 (69.2%)	5 (83.3%)	1.000
Laparotomy	4 (30.8%)	1 (16.7%)	
**FIGO III + IV**	**(*n* = 10)**	**(*n* = 16)**	
Minimally invasive surgery (MIS)	3 (30%)	9 (56.3%)	0.248
Laparotomy	7 (70%)	7 (43.8%)	

*: Statistically significant based on the chi-square test. *p*: *p*-value for comparing between the studied groups, significant at *p* ≤ 0.05. MIS: minimally invasive surgery. **^FE^**: Fisher Exact test.

**Table 5:** Comparison of histopathological, molecular, and surgical characteristics between 122 women with endometrial cancer in Group I and Group II.

	Group I (*n* = 55)	Group II (*n* = 67)	*p*
**1.** **Histopathological subtype**			
Endometrioid	52 (94.5%)	54 (80.6%)	0.111
Serous	2 (3.6%)	9 (13.4%)	
Carcinosarcoma	1 (1.8%)	2 (3.0%)	
Clear cell	0	2 (3.0%)	
**2.** **Molecular subtype**			
Non-Specific Molecular Profile (NSMP)	26 (47.3%)	36 (53.7%)	0.310
Microsatellite Instability-High (MIS-high)	16 (29.1%)	16 (23.9%)	
p53 mutant	7 (12.7%)	12 (17.9%)	
POLE mutant	1 (1.8%)	2 (3.0%)	
Not analyzed	5 (9.1%)	1 (1.5%)	
**3.** **Grading**			
I	24 (43.6%)	31 (46.3%)	0.308
II	19 (34.5%)	15 (22.4%)	
III	12 (21.8%)	19 (28.4%)	
Dedifferentiated	0	2 (3.0%)	
**4.** **FIGO 2009**			
I	32 (58.2%)	45 (67.2%)	0.091
II	13 (23.6%)	6 (9.0%)	
III	8 (14.5%)	15 (22.4%)	
IV	2 (3.6%)	1 (1.5%)	
**5.** **Sentinel Lymph Node (SLN) Mapping**			
	30 (54.5%)	61 (91.0%)	<0.001 *
**6.** **Total Lymph nodes**			
Minimum–Maximum	1.0–49.0	1.0–29.0	
Median	5.0	5.0	0.916
Interquartile range (IQR)	2.0–24.0	3.0–8.5	

*p*: *p*-value for comparing between the studied groups, significant at *p* ≤ 0.05. *: Statistically significant based on the chi-square test. NSMP: Non-Specific Molecular Profile. MSI-high: Microsatellite Instability-High. SLN: Sentinel Lymph Node.

## Data Availability

The data are contained within the article.
